# Progress of Nanomaterials-Based Photothermal Therapy for Oral Squamous Cell Carcinoma

**DOI:** 10.3390/ijms231810428

**Published:** 2022-09-09

**Authors:** Qin Niu, Qiannan Sun, Rushui Bai, Yunfan Zhang, Zimeng Zhuang, Xin Zhang, Tianyi Xin, Si Chen, Bing Han

**Affiliations:** 1 Department of Orthodontics, School and Hospital of Stomatology, Peking University, Beijing 100081, China; 2 National Engineering Laboratory for Digital and Material Technology of Stomatology, Beijing Key Laboratory of Digital Stomatology, Beijing 100081, China

**Keywords:** oral squamous cell carcinoma, photothermal therapy, photothermal conversion agents, nanomaterials, drug delivery system

## Abstract

Oral squamous cell carcinoma (OSCC) is one of the top 15 most prevalent cancers worldwide. However, the current treatment models for OSCC (e.g., surgery, chemotherapy, radiotherapy, and combination therapy) present several limitations: damage to adjacent healthy tissue, possible recurrence, low efficiency, and severe side effects. In this context, nanomaterial-based photothermal therapy (PTT) has attracted extensive research attention. This paper reviews the latest progress in the application of biological nanomaterials for PTT in OSCC. We divide photothermal nanomaterials into four categories (noble metal nanomaterials, carbon-based nanomaterials, metal compounds, and organic nanomaterials) and introduce each category in detail. We also mention in detail the drug delivery systems for PTT of OSCC and briefly summarize the applications of hydrogels, liposomes, and micelles. Finally, we note the challenges faced by the clinical application of PTT nanomaterials and the possibility of further improvement, providing direction for the future research of PTT in OSCC treatment.

## 1. Introduction

For centuries, cancer has been one of the most devastating diseases worldwide [1]. Oral squamous cell carcinoma (OSCC) is associated with high morbidity, high malignancy, and poor prognosis, and has been one of the top 15 most prevalent cancers worldwide [2,3]. However, the current main treatment methods for OSCC (e.g., surgery, chemotherapy, radiotherapy, and combination therapy) have some disadvantages [4]. More specifically, surgery cannot accurately remove all tumor cells, which might lead to cancer recurrence, and the trauma caused by surgery has a significant negative impact on oral function and facial beauty [4,5]. Chemotherapy can cause bone marrow suppression, hepatorenal toxicity, and other severe side effects [6,7,8,9], such as the confirmed cardiotoxicity of doxorubicin (DOX) [10]. Radiotherapy can cause fatigue, hair loss, and gastrointestinal reactions [8,11]. Hence, it is important to develop an innovative approach with improved potency and safety for effective OSCC treatment. Photothermal therapy (PTT) has great development potential because it is less harmful to adjacent normal tissue, induces a lower recurrence rate, has a strong ability to kill tumor cells, and has fewer side effects [12,13].

Hyperthermia has a long history: in Egypt more than 5000 years ago, heat treatment for breast cancer was performed and is recorded in the Edwin Smith Papyrus [14,15]. However, it was not until 1966 that Goldman successfully ablated melanoma with heat generated using a laser, marking the start of PTT history [16,17]. PTT, an emerging and promising treatment modality, employs photothermal conversion agents (PTAs) to generate sufficient heat under near-infrared (NIR) light irradiation to ablate tumor cells [18,19]. It was reported that temperatures as high as 41 °C can cause vasodilatation and increased blood perfusion of cancer tissues, and cause heat shock response of cells. Temperatures above 46 °C can cause irreversible cell damage [20,21]. NIR light is the major wavelength used by lasers in PTT because of its superior tissue penetration ability, lower absorption and scattering in biological tissue, and remote-control ability [19,22]. Moreover, it has high-resolution adjustability in both time and space, such that precise control can be achieved [23]. PTAs are the critical component in PTT and the media to convert the light energy to heat energy. PTAs are injected into the human body and employ targeted recognition technology to accumulate at the tumor site. Under the irradiation of NIR light, light energy is converted into heat energy, raising the temperature of the lesion area to kill cancer cells [24]. An ideal PTA possesses the following features: (i) Excellent photothermal conversion efficiency (PCE); good photothermal conversion performance which is conducive to ablate the tumor and improve treatment efficiency; (ii) good biodegradability, rapid degradation and excretion from human body of PTAs can reduce the potential side effects; (iii) good biocompatibility, PTAs should have low toxicity or even be non-toxic to human cells without NIR irradiation; (iv) and ease of modification, other molecules can be modified on their surface to endow PTAs with additional functions, such as a targeting function [19,25].

Moreover, the rapid development of nanotechnology in recent years provides new ideas for tumor treatment [26,27]. Nanoparticles are much smaller than cancer cells and can easily cross cell barriers. In addition, because of the enhanced permeability and retention (EPR) effect caused by the hyperpermeability of micro-vessels in tumor tissues and the imperfect lymphatic drainage system, nanoparticles can preferentially accumulate in tumor sites to exert their effect [28,29]. Under such circumstances, PTT based on nanomaterials has attracted widespread attention.

**Figure 1:**
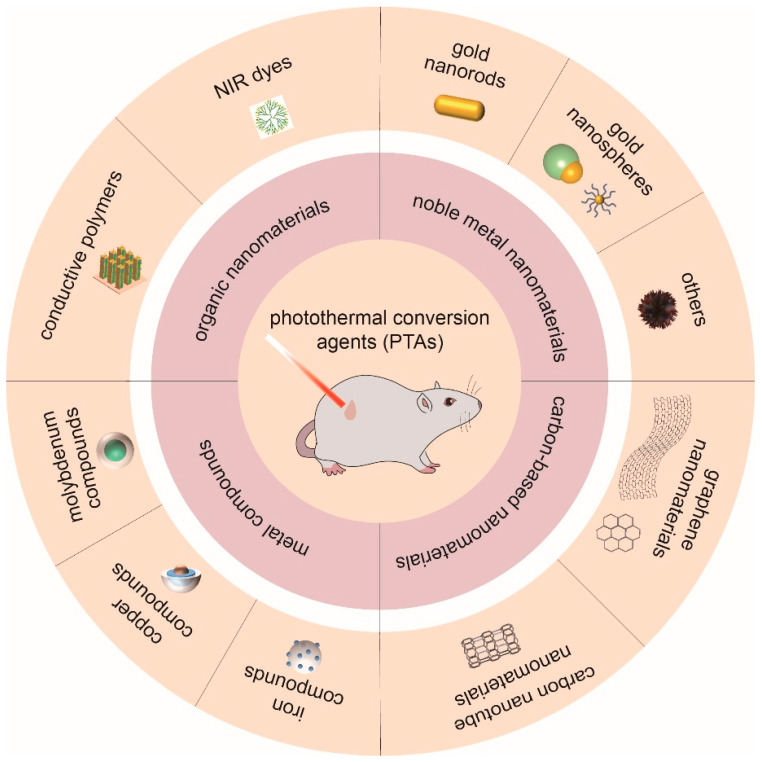
Classification and delivery systems of nanomaterials for photothermal therapy (PTT) in oral squamous cell carcinoma (OSCC).

To date, progress in the application of nanomaterial-mediated PTT in cancer treatment has been introduced in detail in some reviews [12,19,25,30]. However, there has been no review focusing on the field of OSCC. Therefore, this review aims to introduce the progress in the study of nanomaterial-mediated PTT in OSCC. Considering the development history and physicochemical properties of nanomaterials, we divide nanomaterials for PTT in OSCC into four categories: Noble metal nanomaterials, carbon-based nanomaterials, metal compounds, and organic nanoparticles (Figure 1). The synthesis, applications, and therapeutic efficacy in OSCC of the nanomaterials under each classification are introduced in detail. Meanwhile, we briefly summarize the current carriers of PTAs, point out the existing problems of PTT, and discuss future prospects.

## 2. Noble Metal Nanomaterials

**Figure 2:**
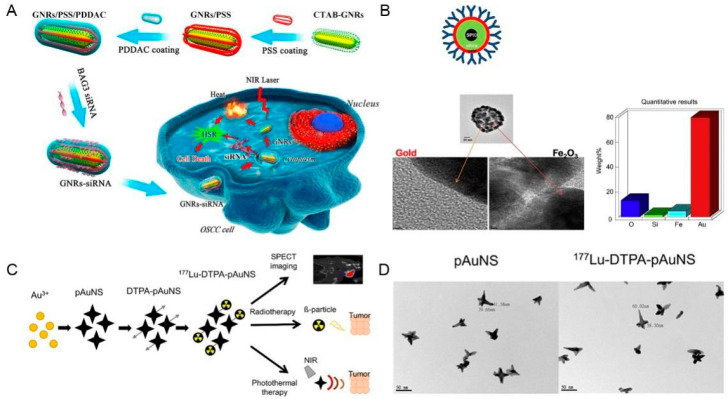
Schematic illustrations of several noble metal nanomaterials. (**A**) Schematic illustration of the design and application of gold-nanorods-small interfering RNA (GNRs-siRNA). (**B**) Structure illustration and high-resolution transmission electron microscopy (TEM) images of C225-ultrasmall paramagnetic iron oxide@Au nanoshell (C225-SPI@Au NS). (**C**) Schematic illustration of the preparation and application of ^177^Lu labeled PEGylated gold nanostars (^177^Lu-DTPA-pAuNS). (**D**) TEM images of pAuNS and ^177^Lu-DTPA-pAuNS. Adapted with permission from [39,40,41]. Copyright 2022, Elsevier, Elsevier and Multidisciplinary Digital Publishing Institute, respectively. PSS: polystyrene sulfonate; PDDAC: polydiallyl dimethyl ammonium chloride; CTAB: Cetyltrimethylammonium Bromide; NIR: near-infrared; OSCC: Oral squamous cell carcinoma; DTPA: Diethylenetriamine pentaacetate; SPECT: single-photon emission computerized tomography; PEG: polyethylene glycol.

The principle of noble metal nanomaterials in PTT is considered to be the localized surface plasmon resonance (LSPR) effect. When the frequency of incident light (usually NIR light) matches the resonance frequency of free electrons in PTAs, PTAs have a strong absorption effect for this part of light, termed the LSPR effect [25,31]. The absorbed photon energy is mainly released and utilized in the form of heat, resulting in the so-called photothermal effect. Generally, noble metal nanomaterials (such as Au, Ag, Pt, and Pb nanomaterials) have an excellent LSPR effect and surface functional modification activity [32,33,34,35]. Surface modification can be carried out by various means to obtain stronger PCE, tumor targeting ability, and better biocompatibility. For example, noble metal nanoparticles can combine with the biocompatible molecule polyethylene glycol (PEG) to increase its water solubility, reduce its immunogenicity in organisms, and increase its time in the systemic circulation [36,37]. Nevertheless, high cost, poor photothermal stability, poor biodegradability, and especially the poor biological metabolism of noble metal nanomaterials cannot be ignored. Moreover, the effects of chronic low toxicity on the human body need to be determined through long-term observation (Figure 2) [25,38].

Au, Ag, Pt, and Pb nanomaterials are extensively applied in PTT for the treatment of lung [42], liver [43,44], breast [45,46], and oral cancer [47,48]. However, in OSCC treatment, gold nanoparticles are one of the most explored and promising PTT nanomaterials; therefore, this review focuses on gold nanoparticles. As in all noble metal nanomaterials, Au nanostructures possess strong LSPR and EPR effects, and can mediate outstanding photothermal conversion [49,50]. PTT mediated by gold nanoparticles can reduce the viability of tumor cells and inhibit the proliferation and migration of tumor cells. Liao et al. combined cysteine functionalized alginate with gold nanorods (GNRs) for PTT, which can reduce the tumor volume by five times in mouse xenograft [51]. In another experiments, integrin-targeting gold nanoparticles showed greater inhibitory effect on the migration of tumor cells [52]. In addition, the PCE of gold nanoparticles for photothermal therapy is determined by their size and morphology [53]. Au nanostructures can be used for PTT in OSCC with different morphologies including nanorods, nanostars, and nanoflowers (Table 1). 

### 2.1. Gold Nanorods

Compared with other gold nanoparticles, because of their adjustable surface plasmon resonance wavelength (by adjusting the aspect ratio), an order of magnitude higher per micron absorption and scattering coefficients, excellent biological imaging function, easier surface functional modification, and colloidal stability, GNRs have been the most widely used gold nanoparticles for PTT in tumor treatment, and the same is true in OSCC treatment [68,69]. The elongated structure of GNRs is conducive to their aggregation in tumor lesions and easily entry into cells [25,70]. Interestingly, in in vitro experiments, Wang et al. selected an oral epidermoid carcinoma cell line (OECM-1) to compare the photothermal properties of gold nanorod composites (up-converting phosphor (UCP)@SiO_2_-NR–folic acid (FA)) with gold nanoparticle composites (UCP@SiO_2_-NP-FA). The unique anisotropic properties and large surface area of GNRs meant that under 980 nm laser irradiation UCP@SiO_2_-NR-FA showed a more outstanding PCE and cancer cell killing effect than UCP@SiO_2_-NP-F. Therefore, UCP@SiO_2_-NR-FA is a more efficient photothermal material [54]. To overcome the cellular defense mechanism of heat shock in nanomaterial-mediated PTT, Wang et al. prepared a gold-nanorods-small interfering RNA (GNRs-siRNA) platform with gene silencing ability to improve the efficiency of PTT. In vivo and in vitro experiments revealed that the siRNA oligonucleotides can be delivered to human cancer cells, where they target and downregulate BAG3 expression, which encodes BAG Cochaperone 3, to prevent the heat shock response. Therefore, GNRs-siRNA nanocomposites can enhance the sensitivity of cancer cells to hyperthermia and achieve efficient PTT. In addition, inhibition of *BAG3* expression enhances the sensitivity of cancer cells to chemotherapy and radiotherapy, which provides a platform for synergistic treatment using PTT, chemotherapy, and radiotherapy (Figure 2A) [39]. Coating GNRs with polyelectrolytes is considered an effective method to make them biocompatible for PTT of cancer. In a study, researchers compared the cytotoxicity to human oral cancer cells (NT8e), PCE, and cellular uptake of different coated GNRs: Polyelectrolyte polystyrene sulfonates-GNRs (PSS-GNRs) and PSS plus poly diallyl dimethyl ammonium chloride-GNRs (PDDAC-GNRs). The results confirmed that PDDAC-GNRs had a higher PCE and cell uptake rate than PSS-GNRs; however, the high cell uptake rate adversely affects its photothermal performance. Therefore, PDDAC-GNRs did not show superior cytotoxicity to NT8e [47].

To realize the targeted PTT of OSCC and achieve the minimum combination of gold nanomaterials with healthy tissues, researchers often combine gold nanomaterials with antibodies, integrins, or cancer cell membranes. GNRs are combined with anti-epithelial growth factor receptor (EGFR) antibody to achieve targeted PTT of OSCC. El-Sayed and coworkers incubated two OSCC cell lines (HSC 313 and hoc 3 clone 8) and a benign epithelial cell line (HaCaT) with GNRs combined with anti-EGFR antibodies, and then exposed them to an 800 nm laser. Subsequently, the authors found that malignant cells needed less than half the laser energy of benign cells and confirmed that the nanomaterial was a safe, potent, and selective PTA [48]. In addition, researchers linked MUA (11-mercaptoundecanoic acid)-capped GNRs with low-molecular-weight chitosan oligosaccharide and then the chitosan oligosaccharide-modified GNRs (CO-GNRs) were further conjugated with an anti-EGFR antibody to developed novel targeted nanomaterial CO-GNRs/anti-EGFR. EGFR was overexpressed in human oral adenosquamous cell carcinoma cell line (Cal-27), thus providing a local targeting function. To confirm the targeting ability of CO-GNRs/anti-EGFR, the material was intravenously injected into animal models with solid tumors. The higher uptake of CO-GNRs/anti-EGFR in the tumor and low uptake in liver demonstrated the targeted delivery of CO-GNRs/anti-EGFR, which was also confirmed by the elevated temperature of tumor tissue under the 780 nm laser irradiation [55]. Recently, Sun and coworkers prepared GNRs coated with cancer cell membrane (GNR@Mem), which is a multifunctional nanoplatform combining PTT and radiotherapy. The cancer cell membrane enhanced the biocompatibility of nanomaterials and had a targeted effect on specific cancer cells, while the GNRs induced a temperature increase and reactive oxygen species (ROS) production under the second NIR window and X-ray irradiation, and then destroyed the DNA spiral structure, resulting in enhanced tumor cell apoptosis [56]. 

To improve the treatment efficiency of oral cancer, some researchers have realized the combined treatment of image-guided PTT, chemo-PTT, and radio-PTT. In addition, the combination of PTT and photodynamic therapy (PDT) is also widely applied in the treatment of OSCC. Wang and coworkers conjugated GNRs with rose bengal (RB) to prepare multifunctional RB-GNRs. They had high photothermal efficiency under 810 nm NIR light and efficient singlet oxygen generation under 532 nm light. In vitro and in vivo, RB-GNRs showed significant anticancer effects and therapeutic advantages. The PDT-PTT combined effect of RB-GNRs in vivo produced better therapeutic effect than PDT or PTT alone, and RB had specificity for oral cells. RB-GNRs, as a multifunctional treatment platform, has great application potential in the treatment of OSCC [57]. Yeo et al. developed a multifunctional cancer treatment nanoparticle based on GNRs coated with the photosensitizer dihydroporphyrin e6 (Ce6) and chemotherapeutic drug DOX on its endogenous human serum (HS) protein corona (NR-HS-Ce6-DOX) [58]. NR-HS-Ce6-DOX can achieve the combination of PDT, PTT, and chemotherapy upon irradiation with a single 665 nm laser to effectively kill Cal-27 cells. In particular, the triple therapy had a strong synergistic effect in killing cancer cells, and the therapeutic effect was better than that of single therapy [58]. In addition, Yeo et al. showed that with a very low dose (50 pM) of NR-HS-Ce6, combined therapy can also achieve almost complete eradication of cancer cells (95.2% cell killing) [59]. Moreover, Zeng et al. synthesized DOX-GNRs@mSiO_2_-HA nanoparticles for PTT in OSCC, which employed multifunctional hyaluronic acid (HA) to modify GNRs/mesoporous silica-based nanoparticles loaded with DOX for photoacoustic imaging (PAI)-guided synergistic chemophotothermal therapy [60]. In vivo, the nanomaterials were injected into animal models for 8 h and exposed to an 808 nm laser. The authors reported a significant difference in the temperature rise at the tumor site between the phosphate buffered saline (PBS) group and the experimental group. In addition, the tumor volume growth rates of the PBS group, chemotherapy only group, and the PTT only group during the experiment were significantly higher from that of the experimental group. A series of results showed that DOX-GNRs@mSiO2-HA had excellent photothermal properties and can be applied for effective synergistic therapy [60].

### 2.2. Gold Nanospheres

Gold nanospheres (GNSPs) have a strong LSPR effect and excellent PCE. Moreover, compared with GNRs, the synthesis method of GNSPs is simpler and safer, and biologically toxic Cetyltrimethylammonium Bromide (CTAB) is not used. Prepared by the citrate reduction method, GNSPs of different sizes can be obtained only by changing the concentration of citric acid and GNSPs can easily combine with various ligands (such as antibodies and DNA). However, the typical absorption band of GNSPs ranges from 500 to 550 nm [71,72]. Although the absorption band can show a red shift with the increase in nanoparticle size, it does not have an adjustable absorption peak in the NIR region. Therefore, its derivatives, hollow gold nanospheres (HAuNSs), are preferred for clinical application. HAuNSs are small in size and have a strong adjustable absorption peak in the NIR region by adjusting the outer diameter and shell thickness. In addition, they have a large internal space and can carry anti-tumor drugs to achieve synergistic therapy [61,62,63,64,65,66,67,68,69,70,71,72,73,74]. 

GNSPs have strong absorption at 500–550 nm in the visible region and weak absorption in the NIR region. However, two photons at the NIR region were converted into one photon at the visible region in the second harmonic generation, which enabled GNSPs to absorb NIR light. Huang et al. fabricated anti-EGFR/Au conjugates by combining GNSPs with an anti-EGFR antibody, which realized the targeted combination to HSC oral cancer cells. Subsequently, they exposed cells treated with anti-EGFR/Au conjugates to the 800 nm laser, with a pulse duration of 100 femtoseconds. The experimental result showed the laser power threshold of destruction of cells treated with anti-EGFR/Au conjugates was 20 times lower than that required for cell destruction in normal PTT [74]. EGFR is overexpressed in 90% of head and neck cancer cells; therefore, it has been widely studied and applied in the field of head and neck cancer targeted therapy. Melancon et al. compared the selective EGFR targeting ability of aptamer-HAuNSs, PEG-HAuNSs, and antibody-HAuNSs (C225-HAuNSs). In vitro and in vivo experiments demonstrated that the aptamer was a promising ligand and can achieve efficient targeted treatment for HNSCC [61]. 

C225-ultrasmall paramagnetic iron oxide@Au nanoshell (C225-SPIO@AuNS), fabricated by Melancon and coworkers, can achieve imaged-guided targeted PTT. It was confirmed in vitro and in vivo that it can selectively bind head and neck cancer cell lines overexpressing EGFR (HN5, FaDu, and OSC-19) and human squamous carcinoma A431 cells, which also overexpressed EGFR. An in vitro photothermal ablation experiment showed that the cells treated with C225-SPIO@AuNS experienced significant cell killing under NIR light irradiation at 808 nm and C225-SPIO@AuNS demonstrated its selective binding and selective photothermal ablation ability. Moreover, the nanocomposite turned out to be magnetic resonance (MR)-active and can assist PTT for tumors in clinical application (Figure 2B) [40]. 

### 2.3. Others

Branched gold nanostructures, such as gold nanoflowers (GNFs) and gold nanostars (GNSTs), have sharp edges and unique branch angle structures. This unique morphology endows them with unique optical properties. By changing the core size, number, length, and width of the sharp branches, the absorption band of branched gold nanostructures can be adjusted to the NIR region. In addition, branched gold nanostructures have a large surface area, and the “hot spots” at their sharp corners greatly enhance the intensity of the surrounding electromagnetic field, endowing them with a large surface enhanced Raman scattering (SERS) effect and broad application prospects [75,76,77].

Recently, a new type of GNF particle was synthesized using the aqueous seed method. The prepared GNF particles had an absorption peak at 719 nm and had efficient photothermal conversion under the NIR irradiation. The results showed that the prepared GNF particle-mediated photothermal therapy can significantly inhibit the proliferation of tongue cancer cells (TCA-8113), and the relative growth rate of TCA-8113 cells decreased with increasing GNF concentration and laser power [62]. Similarly, Song et al. prepared gold nanoflower double-layer silica core-shell nanoparticles and studied their photothermal properties and in vivo biological toxicity [63]. In other study, Lin et al. combined the β^−^-emitter ^177^Lu with Diethylenetriamine pentaacetate-PEG decorated gold nanostars (^177^Lu-DTPA-pAuNS) to treat HNSCC. In a human HNSCC tumor-bearing animal model, comparisons among the control group, the ^177^Lu-DTPA-injected group, and the ^177^Lu-DTPA-pAuNSs-injected group supported the excellent accumulation and brachytherapy effect of the ^177^Lu-DTPA-pAuNS in tumors. The researchers also found that the survival rate of tumor-bearing mice in the ^177^Lu-DTPA-pAuNSs-injected group was significantly higher than that in the other groups after 20 days. Moreover, compared with ^177^Lu-DTPA-pAuNS alone, the combination of ^177^Lu-DTPA-pAuNS and PTT (793 nm, 1 W/cm^2^, 6 min) can further enhance the tumor inhibition effect. Therefore, ^177^Lu-DTPA-pAuNS has great application prospects in the field of synergistic therapy of brachytherapy and PTT (Figure 2C,D) [41]. In addition, Sasidharan et al. revealed that gold nanostars have good computed tomography contrast characteristics and obvious photothermal cytotoxicity to oral epithelial cancer cells [64]. 

Mapanao et al. developed a gold nanostructure (tNAs-cisPt) composed of gold ultrasmall nanoparticles and an endogenously double controlled cisplatin prodrug for combined chemo-photothermal treatment of HNSCC. The nanostructure tNAs-cisPt can achieve the combined treatment of chemotherapy and PTT, and the experiment showed that compared with chemotherapy alone (89.0 ± 6.0%) or PTT alone (109.3 ± 5.0%), combined therapy can reduce the cell survival rate to 78.3 ± 1.2%. In addition, compared with that against Human papilloma virus (HPV)-negative HNSCC, combination therapy had more significant antitumor effect on HPV-positive HNSCC [65]. Sasidharan and colleagues developed a multifunctional nanoplatform of blood and cell compatible branched chain gold nanostructures based on protein nanoparticles, which can be used for dual CT diagnosis and photothermal therapeutic agents [66].

In addition to gold nanomaterials, other noble metal nanomaterials such as silver, platinum, palladium, and Au-Ag nanoparticles are also widely used in PTT of tumors. However, there is little research on their application in the field of OSCC. Su et al. developed a new protein hybrid hydrogel nanoparticle (E_72_-Chitosan-Ag_3_AuS_2_) for PTT in situ tongue tumors. The as-prepared soft material possessed a network formed by negatively charged proteins, chitosan molecules, and Ag_3_AuS_2_ NPs, and exhibited good biocompatibility, excellent biodegradation, few side effects, and excellent PCE because of its structure. In vivo, PTT mediated by the E_72_-Chitosan-Ag_3_AuS_2_ hydrogel nanomaterial can significantly eliminate the sublingual carcinoma cells of tumor-bearing mice. After treatment, no obvious damage or abnormality was found in the main organs, such as the heart, liver, spleen, lung, and kidney. Thus, the E_72_-Chitosan-Ag_3_AuS_2_ hydrogel nanomaterial held great potential for clinical application of PTT in OSCC [67]. 

## 3. Carbon-Based Nanomaterials

**Figure 3:**
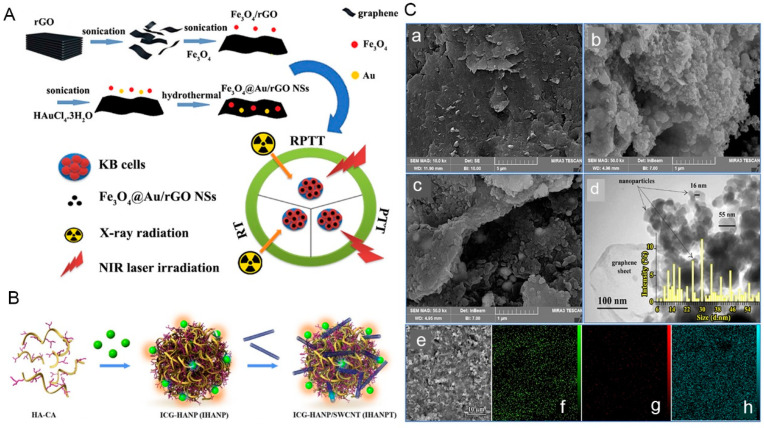
Schematic illustrations of several carbon-based nanomaterials. (**A**) Schematic illustration of the synthesis process and applications of Fe_3_O_4_@Au/reduced graphene oxide nanostructures (Fe_3_O_4_@Au/rGO NSs). (**B**) Schematic illustration of the synthesis process of Indocyanine Green (ICG)-coupled threadlike nanoparticles (IHANPT). (**C**) Field emission scanning electron microscope (FE-SEM) images of (**a**) GO and Fe_3_O_4_@Au/rGO NSs with (**b**) 10 and (**c**) 40 wt%, and (**d**) TEM image and particle size distribution of Fe_3_O_4_@Au/rGO NSs with 10 wt% rGO content. (**e**) Scanning electron microscope (SEM) image of Fe_3_O_4_@Au/rGO NSs with 40 wt% and corresponding EDS elemental mapping images of (**f**) C, (**g**) Au, and (**h**) Fe acquired at the same region. Adapted with permission from [81,82]. Copyright 2022, Elsevier and American Chemical Society, respectively. EDS: energy dispersive spectroscopy; RPTT: radio-photothermal therapy.

Carbon-based nanomaterials have poor NIR light absorption ability and dispersion in water; however, they have excellent electrochemical properties, strong non-covalent bonding properties, and a large surface absorption area. Appropriate surface functional activity modification, such as surface-linked PEG or encapsulated polymers, not only increase their dispersion ability in water and photothermal conversion efficiency, but also increase their drug loading ability, which can realize the cooperation of PTT and chemotherapy for tumors. Carbon is also the basic element of the human body; therefore, the biocompatibility of carbon-based nanomaterials is significantly better than that of other materials (Figure 3, Table 2) [78,79,80].

### 3.1. Graphene Nanomaterials

Graphene possesses excellent physical, chemical, and structural properties, such as ballistic conductivity, high elasticity, large surface area, and rapid heterogeneous electron transfer, which has attracted great interest of researchers in the field of biological applications [80]. In particular, graphene shows a significant photothermal effect because of its high absorption in the NIR region, which opens up a new direction for PTT of tumors. 

The therapeutic effect of radio-photothermal therapy on the KB human OSCC cell line has been studied by researchers. Ardakani et al. synthesized Fe_3_O_4_@Au/reduced graphene oxide (rGO) nanostructures (NSs) at different concentrations (10 and 40 wt%) for the radio-photothermal therapy of KB cells. In experiments, upon exposure to 808 nm laser light (1.8 W/cm^2^), Fe3O4@Au/rGO NSs (40 wt%) had a high PCE (up to 61%). In addition, upon exposure to 808 nm light and 4 Gy X-ray, Fe_3_O_4_@Au/rGO NSs with 40 wt% rGO at a concentration of 20 ug/mL displayed strong cell destruction ability (88.1%), showing that the effect of radio-photothermal therapy is better than that of PTT and radiotherapy alone (Figure 3A,C) [81]. In other work, Gao et al. synthesized a fluorescent and photoacoustic (PA) imaging guided enhanced PTT nanomaterial. Researchers seeded gold nanoparticles onto graphene oxide. Furthermore, NIR dye Cy5.5 labeled-matrix metalloproteinase-14 substrate was conjugated onto the graphene oxide complex forming an improved smart theranostic probe CPGA. After CPGA was injected intravenously into SCC7 tumor-bearing mice, subsequently high fluorescence and PA signals could be observed in the tumor area, reaching a peak at 6 h after injection. Under irradiation of an 808 nm laser (0.75 W/cm^2^), excellent tumor inhibition without recurrence was observed at 6 h after injection [83].

### 3.2. Carbon Nanotubes

Carbon nanotubes are reported to have excellent electrical, mechanical, and thermal properties because of their nanostructures. In addition, their NIR absorption characteristics and the ability to convert light energy into heat energy mean that carbon nanotubes and modified carbon nanotubes are often used as photothermal agents [92,93]. 

Wang et al. combined indocyanine green (ICG) with hyaluronic acid nanoparticles encapsulated by single wall carbon nanotubes (SWCNTs) to prepare a new therapeutic nanoplatform termed ICG-HANP/SWCNT (IHANPT). This nanoplatform is a CD44-targeted and PA imaging-guided PTT and PDT combined phototherapy agent for cancer treatment. In a human tumor xenograft model, the CD44 targeting effect of the nanoplatform led to significant accumulation of IHANPT in the tumor tissue. Under laser irradiation (808 nm, 0.8 W/cm^2^), ICG molecules can produce ROS to inhibit tumor through PDT. Meanwhile, the PTT effect resulting from ICG and SWCNT can increase the tumor temperature to 55.4 ± 1.8 °C, inhibit tumor growth, and induce tumor cell death. No obvious systemic and local toxic effects were found in the mice (Figure 3B) [82].

In addition to graphene and carbon nanotubes, other types of carbon-based nanomaterials are also used in PTT of OSCC. Das et al. developed uniform-sized (65–70 nm) N-rich mesoporous carbon nanospheres (NCOD-HCS). These carbon spheres contain a void space, in which ultra-small nitrogen-doped quantum dots (NCQDS) are captured. In vitro experiments in human oral cancer cells (FaDu) demonstrated that NCQD-HCS had favorable photothermal properties. It was found that the temperature of the NCOD-HCS solution at a concentration of 2 mg/mL would increase to 51 ℃ after exposure to a 980 nm laser for 15 min. After 5 min of 980 nm laser irradiation, the survival rate of FaDu cells treated with NCQD-HCS decreased significantly [84]. 

## 4. Metal Compounds

**Figure 4:**
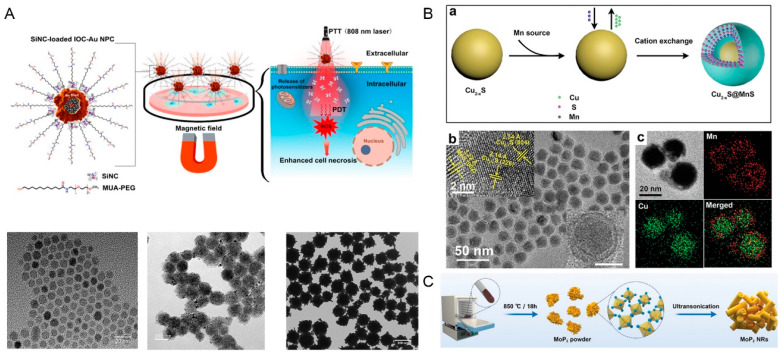
Schematic illustrations of several metal compounds. (**A**) Schematic illustration of gold nanopopcorns containing a self-assembled iron oxide cluster core coated with near-infrared-absorbing photosensitizer silicon 2,3-naphthalocyanine dihydroxide and stabilization with poly (ethylene glycol) linked with 11-mercaptoundecanoic acid (MUA-PEG/SiNC/IOC-Au NCPs) and TEM images of IO NPs, IOCs, and IOC–Au NPCs. (**B**) Synthesis scheme and TEM morphology of Cu_2−x_S@MnS core-shell nanoparticles (Cu_2__−x_S@MnS CSNPs). (**a**) Synthesis scheme of Cu_2*−x*_S@MnS CSNPs. (**b**) TEM morphology of as-synthesized CSNPs. Inset in the upper left corner: high-resolution images. Inset in the lower right corner: TEM image of a single CSNP. (**c**) EDS elemental mapping of Cu_2*−x*_S@MnS CSNPs. (**C**) Morphology and characterization of as-synthesized molybdenum diphosphate nanorods (MoP_2_ NRs). Adapted with permission from [86,88,90]. Copyright 2022, John Wiley and Sons, American Chemical Society and John Wiley and Sons, respectively.

Metal compounds have good biocompatibility and high PCE; therefore, they are considered promising materials for PTT of OSCC. Compared with noble metal nanomaterials, other metal compounds have attracted much attention because of their low cost, good photothermal stability, and low cytotoxicity. In addition, metal compounds can be used as drug carriers, which creates conditions for the combination of photothermal therapy and other therapies, and the preparation of multifunctional nanoplatforms (Figure 4, Table 2) [94,95].

### 4.1. Iron

Fe_3_O_4_ nanoparticles can induce high temperature under alternating magnetic field (AMF) because of their unique magnetism. The heat generated by magnetic hyperthermia is enough to destroy cancer cells. Fe_3_O_4_ nanoparticles can also induce hyperthermia under NIR laser radiation. However, magnetic hyperthermia may affect healthy tissues and photo hyperthermia has certain safety advantages [50,96,97].

Shen et al. prepared a kind of multifunctional magnetic therapeutic modality with a drug targeted delivery capability, low cellular toxicity, high PCE, and a simple structure. Fe_3_O_4_ particles were modified by Carboxymethyl Chitosan (CMCTS) to obtain stable Fe_3_O_4_@CMCTS particles. In vivo, by utilizing the magnetism of Fe_3_O_4_ nanoparticles and attaching a magnet to the tumor, MR imaging revealed that Fe_3_O_4_@CMCTS particles tended to accumulate in the tumor and showed excellent drug targeted delivery capability. In addition, after exposure to an 808 nm laser (1.5 W/cm^2^) for 5 min, the tumors in the Fe_3_O_4_@CMCTS + laser group can be destroyed by high temperature (up to 52 °C) generated by PTT. In the meantime, there was little damage to the main organs of the mice [85]. Fe_3_O_4_ particles are commonly applied for magnetic field-guided drug delivery. Bhana et al. developed a new nanomaterial MUA-PEG/SiNC/IOC-Au NCPs (covered iron oxide cluster and gold nanopopcorns) using a seed-mediated growth method. The nanocomposite combined magnetic field-guided drug delivery, PTT, and PDT to treat tumors. In vitro, the combined therapy significantly decreased the survival rates of KB-3-1 (oral cancer) and SK-BR-3 cells (breast cancer) (Figure 4A) [86].

### 4.2. Copper

Copper compound nanomaterials (including copper selenide, copper telluride, and copper oxide) are promising nanomaterials for PTT in OSCC [98]. Among them, copper sulfide nanomaterials are one of the most promising copper compound nanomaterials for PTT of cancer, including OSCC because of their low production cost, low toxicity, small volume, and strong NIR light absorption capacity [99,100]. However, we have little knowledge about their potential toxicity effects. Therefore, Feng et al. evaluated the in vivo and in vitro toxicity of CuS nanoplates for PTT. In vitro studies showed that they had different degrees of inhibition on different cell lines, and the adverse effect on the cytoskeleton may be one of the main reasons. The CuS nanoplates were more easily engulfed by phagocytes than non-phagocytes. In addition, CuS nanoplates were mainly distributed in organs with clear function such as the liver, spleen, and lung [101]. Additionally, Zuo et al. fabricated a multi-functional nanomedicine Cu_2−x_S-RB@DMSN-AE105 (CRDA) for the treatment of OSCC. AE105 targeted the binding the urokinase plasminogen activator receptor overexpressed in OSCC cell membranes, which enabled CRDA to accumulate at tumor sites. Under irradiation of an NIR laser, Cu_2−x_S as PTAs can achieve tumor heat ablation by photothermal conversion. In addition, the sonosensitizer RB can kill OSCC tumor cells via a sonodynamic effect [87]. In other work, Huang et al. developed a new nanosystem based on Cu_2−x_S@MnS core-shell nanoparticles for the combined treatment of PTT and PDT. The Cu_2−x_S core, as a photosensitizer, can produce heat and ROS for PTT and PDT, while the MnS shell can produce O_2_ under the stimulation of H_2_O_2_. In addition, in vivo, both HeLa tumor cell line derived xenograft models and HNSCC patient-derived xenograft models showed the core-shell nanoparticles led to tumor inhibition and can be used as an efficient tumor treatment agent via a combination of PTT and PDT (Figure 4B) [88].

Maor and coworkers encapsulated copper oxide nanoparticles (CuO NPs) in Poly (lactic-co-glycolic acid) (PLGA)/polydopamine (PDA)/PEG nanospheres and developed two drug delivery systems that can realize the control and continuous release of CuO NPs (CuO-NPs@H-PLGA/PDA/PEG and CuO-NPs@L-PLGA/PDA/PEG). The PLGA content of the two drug delivery systems was different. In vitro, experiments demonstrated that upon exposure to an 808 nm laser, the CuO NPs release rate of CuO-NPs@L-PLGA/PDA/PEG was much faster than that of CuO-NPs@H-PLGA/PDA/PEG, which also led to a stronger antitumor effect in a head and neck cancer cell line (Cal-33) [89].

### 4.3. Molybdenum

Molybdenum is a transition metal, and its compounds have also been explored as photosensitizers [102]. Chemodynamic therapy is a treatment modality that employs chemical agents to decompose hydrogen peroxide (H_2_O_2_) into hydroxyl radical by Fenton or Fenton-like reactions, which induces cell apoptosis or necrosis, and possesses many favorable properties, such as high tumor-specificity and minimal-invasiveness. However, the therapeutic efficiency of chemodynamic therapy is limited by the high pH value of the tumor site. To overcome this difficulty, Qian et al. developed a new nanomaterial, molybdenum diphosphate nanorods (MoP_2_ NRs). Under 808 nm laser irradiation, MoP_2_ NRs can effectively achieve the combination of mild PTT and enhance chemodynamic therapy, which was confirmed in in vivo experiments in a Cal-27 oral tumor model in nude mice (Figure 4C) [90]. 

In addition, Chen and colleagues developed ideal low toxicity PTAs for the treatment of OSCC by fabricating chiral molybdenum (Cys-MoO_3_-x) NPs, which were subsequently modified by cysteine molecules. Cys-MoO_3_-x NPs had dual photothermal effects in the visible region and NIR region. In vitro, Cys-MoO_3_-x NPs significantly reduced the survival rate of OSCC cells, and the cell death rate caused by PTT increased with the increase in the laser wavelength from 405 to 808 nm. The study developed potent and low toxicity PTAs and showed a great application prospect in the PTT treatment of OSCC [91].

## 5. Organic Nanoparticles

**Figure 5:**
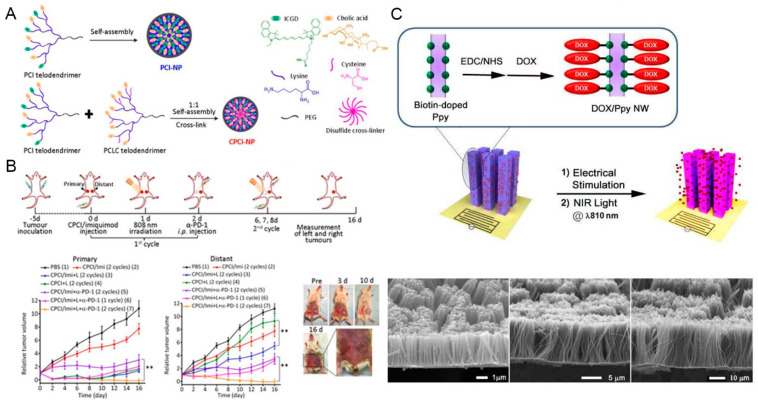
Schematic illustrations of several carbon-based nanomaterials. (**A**) Schematic self-assembly illustration of photochemical internalization nanoparticle (PCI−NP) and the coassembled nanoparticle (CPCI−NP). (**B**) Synergistic anti-tumor activity of photothermal-/immunotherapy in the mice bearing orthotopic 4T1 breast cancer (both sides). ** *p* < 0.01 in the line chart on the left by comparing group 5 (CPCI/Imiquimod−NP + α−PD−1) with group 7 (CPCI/Imiquimod−NP +808 nm laser + α−PD−1); ** *p* < 0.01 in the line chart on the right by comparing group 5 (CPCI/Imiquimod−NP + α−PD−1) with group 7 (CPCI/Imiquimod−NP + 808 nm laser + α−PD−1) and comparing group 4 (CPCI/Imiquimod−NP) with the group 3 (CPCI/Imiquimod−NP + 808 nm laser). (**C**) Schematic illustration of DOX−attached polypyrrole (Ppy) nanowires (DOX/Ppy NWs) and SEM images of the fabricated Ppy NW arrays. Adapted with permission from [104,105]. Copyright 2022, American Chemical Society. ICGD: indocyanine green derivative; EDC: Ethyl−3−(3−Dimethylaminopropyl); NHS: N−hydroxysuccinimide; αPD−1: alpha programmed cell death; 1PBS: phosphate-buffered saline.

In addition to inorganic nanomaterials, organic nanomaterials are also promising materials for PTT of oral cancer. Organic nanomaterials can be used as photothermal agents because their large π-conjugated system endows them with NIR light absorption properties [103]. In addition, organic nanomaterials are photothermal agents composed of natural substances in organisms. Therefore, they have excellent biocompatibility and biodegradability, and can avoid the adverse reactions caused by the long-term retention of foreign matter in the body [19,103]. At present, organic PTT agents for oral cancer mainly include NIR dyes and conductive polymer nanomaterials (Figure 5, Table 3). 

### 5.1. NIR Dyes

Cyanine and porphyrin derivatives are common NIR dyes for PTT. Among them, indocyanine green (ICG) is the most common organic nanomaterial for PTT in oral cancer [117]. It is the only organic NIR dye approved by the U.S. Food and Drug Administration for human medical imaging and clinical diagnosis [118]. Many studies have reported the application of ICG-based nanomaterials for PTT of oral cancer. 

Interestingly, ICG can also be used for PDT. Researchers have developed an organic compound (C3) and encapsulated it with ICG in PEG-polycaprolactone (PCL) to form mixed nanoparticles (PEG-PCL-C3-ICG NPs) for PTT and PDT treatment of OSCC. As a new PTT and PDT agent, C3 can generate heat under the irradiation at 808 nm and shows an excellent photothermal conversion ability, lower cytotoxicity, and faster metabolic rate. At the same time, ICG is regarded as a PDT agent, and can produce ROS and fluorescence guiding effects upon irradiation of the tumor site with 808 nm laser. The new nanocomposite is a promising agent for the PTT and PDT combination treatment in OSCC [106]. In other work, Song et al. successfully constructed ICG-loaded gold nanocomposite for the synergistic effect of PDT and PTT in oral cancer. The nanocomposite was formed by gold nanoflower core and double-layer silica shell (AuNFs@SiO_2_@mSiO_2_-ICG) [107]. Ren et al. prepared a multifunctional spherical nanoparticle (DOC-SINPs) containing docetaxel, stromal cell-derived factor 1 (SDF-1), ICG, and perfluorohexane (PFH). The multifunctional nanoparticles, with a diameter of 502.88 ± 17.92 nm, had excellent targeting ability and high PCE. In vitro, the new agent significantly reduced the survival rate of tongue cancer cells (SCC-15) [108]. 

Zhang and colleagues fabricated multifunctional micelle nanosystem CPCI-NP containing PTA ICG derivatives (ICGD). CPCI-NPs are an excellent carrier of DOX. CPCI/DOX-NPs have been proved to be a synergistic efficient PTT and chemotherapy nanoplatform for OSC-3 oral cancer cells. The study showed that more than 87% of OSC-3 cells were killed by CPCI/DOX-NP under laser irradiation (Figure 5A,B) [104]. Xiong et al. developed multifunctional targeted nanoparticles loaded with ICG and DOX (SDF-1/ICG/PFH/DOX/PLGA NPs) for targeted PA imaging and PTT of oral cancer cells. Chemokine SDF-1 achieved enhanced local accumulation in oral cancer SCC-15 cells by specifically binding to C-X-C motif chemokine receptor 4. The experimental results in vivo and in vitro demonstrated that the nanoparticles had good photoacoustic imaging characteristics and photothermal therapy ability [109]. Similarly, Sun and coworkers used PLGA as a carrier and encapsulated the chemotherapeutic agent DOX and photosensitizer ICG in the carrier. Then, chemokine SDF-a was introduced and endowed the new PTA (SDF-a/ICG/PNE/ ADRPLGA NPs) with an excellent targeting ability. The new nanoplatform can be used for PTT under PA guidance of oral cancer cells. In the rabbit tongue cancer lymph node metastasis model, the therapeutic nanoplatform showed an excellent anti-tumor effect [110]. In another study, a new drug delivery system comprising human serum albumin-ICG-cisplatin nanoparticles (HSA-ICG-DDP NPs) was developed. The authors found that the nanosystem had the synergistic effect of PDT, PTT, and chemotherapy on oral cancer [111]. In addition, Shi et al. prepared poly β-Amino ester (PBAE)/PLGA mixed nanoparticle loaded with ICG and an *NRF2* (encoding NF-E2-related factor 2) siRNA. ICG was used as the photosensitizer, and the *NRF2* siRNA was an effective synergist for the amplification of PDT. The mixed nanoparticles were then encapsulated in cancer cell membrane vesicles, which were specially derived from homologous oral tongue squamous cell carcinoma, named M@PPI-siRNA. M@PPI-SiRNA endowed mixed nanoparticles with strong oral tongue squamous cell carcinoma targeting ability. In addition, through the combination of PTT and amplified PDT, the newly synthesized nanoparticles had strong oral tongue squamous cell carcinoma inhibition ability [112]. In other work, Gel-N-ICG nanoparticles were fabricated for combination treatment of PTT and immunotherapy of HNSCCs. Gel-N-ICG nanoparticles were covered with matrix metalloproteinase-degradable gelatin nanoparticles, ICG, and the signal transducer activator of transcription 3 inhibitor NSC74859. NSC74859 can induce effective antitumor immunity [113]. To overcome the problems of a hydrogel drug delivery system, such as inaccurate drug release and limited light-absorption efficiency, recently, a hybrid system was developed by introducing mesoporous silica nanoparticles (MSNs) as DOX carriers into the IR820/methylcellulose hydrogel networks. MSNs can achieve accurate and controlled drug release of DOX. IR820, a new green cyanine dye, is a PTT agent and can realize light-heat conversion under NIR irradiation [114]. 

Moreover, Muhanna et al. proved that porphyrins can not only perform fluorescence and PA imaging of buccal and tongue cancer, but also complete targeted ablation of these tumors. In a buccal carcinoma rabbit model, complete thermal ablation of the tumor can be achieved after 35 days of porphysome-PTT treatment [115]. 

### 5.2. Conductive Polymers

Conductive polymers (such as polypyrrole (Ppy), polyaniline, and poly (3,4 ethylenedioxythiophene)) are more cost-effective than noble metal nanomaterials and have better biocompatibility than inorganic nanomaterials; therefore, conductive polymers as photothermal agents have attracted the attention of researchers [119]. We summarize the application of Ppy as a photothermal agent in the field of oral cancer. 

In the study of Lee et al., DOX conjugated Ppy nanowire (DOX/Ppy NW) arrays were prepared. Ppy NW as an effective drug release system can induce the release of DOX under the influence of an external electric field. PTT mediated by Ppy maximized the chemotherapy effect of DOX. The research in two types of cancer cell line, namely KB cells and human breast cancer MCF7 cells, revealed the potent anti-tumor effect of DOX/Ppy NW. In vitro, the survival rates of KB cells and MCF7 cells treated with DOX/Ppy NW decreased by 70% under the influence of electrical stimulation and NIR laser irradiation [105]. However, the clinical application of conductive polymers was limited due to the lack of safety data. Moreover, conductive polymers have only a small number of reactive groups for functional modification. Therefore, it is important to develop conductive polymer nanoparticles with rich functional groups (Figure 5C). 

In addition, Gu et al. reported that terbium ion-doped hydroxyapatite (HATb) nanoparticles can be used as luminescent probes to encapsulate the NIR photothermal agent PDA and DOX for imaging guided synergistic therapy of chemotherapy and PTT [3]. Xu et al. successfully prepared a new nanosystem for OSCC using HA and hollow mesoporous Prussian blue nanoparticles, which were loaded with DOX (HMPBs&DOX@HAMNs). HMPBs&DOX@HAMNs can heat the tumor tissue, maintain its internal temperature above 60 °C, and regulate the release behavior of DOX. HMPBs&DOX@HAMNs displayed a strong anti-tumor effect in vivo and in vitro [116].

## 6. Drug Delivery System

Conventional PTT cannot achieve targeted delivery of photothermal nanomaterials and has some limitations, for example, PTAs cannot concentrate on the lesion site, resulting in an unsatisfactory treatment effect. Drug delivery systems can realize targeted drug delivery and controlled drug release in cancer treatment, thus improving its effectiveness. Meanwhile, reduced drug accumulation at normal organs can reduce the damage to adjacent normal tissues. In addition, drug delivery systems also have good biocompatibility and biodegradability [120,121,122,123,124]. Therefore, in recent years, researchers have developed many nanostructures such as hydrogels, liposomes, cell membranes, and micelles as drug delivery systems in cancer treatment to improve the treatment effect and reduce side effects. These drug delivery systems are also used in PTT of OSCC.

### 6.1. In Situ Hydrogels

**Figure 6:**
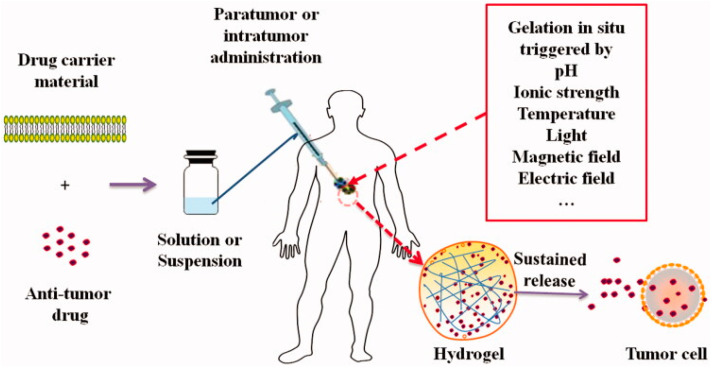
Schematic process of formation of in situ hydrogel and sustained release of drugs from the hydrogel into tumor cells. Adapted with permission from [120]. Copyright 2022, Taylor & Francis.

In situ hydrogels, because of their excellent properties, are regarded as potential biomaterials for cancer therapy, and have become research hotspots in biomedical and pharmaceutics fields. An in situ hydrogel drug delivery system can be administrated into the human body in solution, and the solution-gel phase change occurs immediately in the site of administration. The hydrogels can change from solution or suspension to a semi-solid or solid state [125]. An in situ injectable hydrogel is a three-dimensional network formed by hydrophilic polymers with high drug loading ability and good biocompatibility. The characteristics of the solution-gel phase transition of injectable in situ hydrogel system can achieve targeted delivery of therapeutic drugs, extend the release time of drugs, and increase the concentration and residence time of the drug at the lesion site, thereby maximizing the therapeutic effect to the lesion sites and minimizing the toxicity to normal tissues (Figure 6) [124,125,126]. 

To overcome the limitations of conventional PTT, in situ hydrogel systems can be applied to PTT for oral cancer. As mentioned above, Su et al. prepared an NIR-light responsive injectable hydrogel system for PTT of in situ tongue cancer. This in situ tongue cancer treatment system comprised E_72_-Chitosan-Ag_3_AuS_2_ as a three-dimensional network and its multiple interactions endowed it with injectability and a stable structure. Thus, the hydrogel drug delivery system, with many excellent properties, significantly improved the treatment effect of PTT and enabled in situ tongue cancer to be eliminated by one-time PTT [67].

In situ injectable hydrogel systems are often applied in combination therapy for cancer because of their excellent properties. Chen et al. developed an injectable hydrogel multifunctional treatment system for in situ treatment of cancer, which packaged FeGA and was called FH. Through intratumoral injection, FH can achieve accumulation in the lesion region and stable controlled release of therapeutic drugs. FeGA has a stable photothermal conversion ability and can raise the temperature for therapeutic drug release. At the same time, FeGA can react with hydrogen peroxide in cells to produce hydroxyl radical (Fenton reaction), which can damage cells and reduce radiation resistance. In vitro and in vivo studies showed that the new FH nanosystem could realize the combination of low-dose radiotherapy, chemodynamic therapy, and PTT under NIR and X ray (2 Gy) irradiation [127]. 

In addition, in situ hydrogel systems, with broad application prospects, have great potential not only in the field of cancer treatment, but also in tissue engineering and antibacterial therapy. They can repair tissue defects caused by tumors and tumor residues, and bacterial infections after surgery, which is conducive to tumor treatment [128,129].

### 6.2. Liposomes

Liposomes, spherical bilayer vesicles composed of molecules such as phospholipids and cholesterol in which drugs can be encapsulated, are promising biomaterials for cancer treatment. Liposomes have an aqueous core and a lipid bilayer with hydrophobic properties, which can not only load hydrophilic molecules, but also deliver some hydrophobic molecules that are not suitable for intravenous administration to the lesion site [130]. In addition, because the components of liposomes, such as phospholipids, sphingolipids, and cholesterol, are also components of human cell membranes, they have good biocompatibility and biodegradability, and are easy to be metabolized by the human body. Liposomes can be modified by connecting antibodies and peptides, and these molecules can specifically recognize and bind to specific molecules on the cancer cell membrane, so as to achieve targeted tumor therapy. At the same time, liposomes can increase the aggregation of drugs at the tumor site, realize the controlled release of drugs, and prolong the action time of drugs. These excellent properties enable liposomes to enhance the effectiveness of cancer treatment and reduce the adverse side effects on normal tissues [131,132].

Liposome drug delivery systems can be used for photothermal treatment of oral cancer. As mentioned above, Shen et al. developed multifunctional magnetic nanoparticles loaded with thermosensitive liposomes (Fe_3_O_4_-TSL) and further encapsulated DOX into Fe_3_O_4_-TSL (DOX-Fe_3_O_4_-TSL). This nanomaterial therapeutic platform can realize the combination of PTT and tumor chemotherapy. In addition, liposomes possess great potential in the field of tumor synergistic therapy. Anilkumar and his colleagues prepared magnetic liposome composites by wrapping iron oxide magnetic nanoparticles (CMNPs) in cationic liposomes, which can be used for dual magnetic photothermal cancer treatment induced by AMF and NIR lasers [133].

Xu et al. encapsulated the water-soluble immunostimulatory molecule polyinosinic acid in the hydrophilic core of liposomes and added the organic dye ICG into the lipid bilayer to prepare the thermal response liposome (PITRL) containing polyinosinic acid and ICG. Under NIR irradiation at 808 nm, ICG can convert light energy into heat energy and produce a photothermal effect. In addition, the elevated temperature of PITRL can cause the release of polyinosinic acid, which can induce the activation of dendritic cells in tumor draining lymph nodes. Subsequently, the activation of dendritic cells can prevent the growth of lung metastasis after vein transplantation of cancer cells [134].

### 6.3. Micelles

Micelles are amphiphilic colloidal nanostructures composed of amphiphilic molecules which have a hydrophobic core and a hydrophilic shell. Micelles are considered promising drug delivery systems for cancer treatment because of their excellent properties. The amphiphilic nature of micelles enables the micelles to encapsulate hydrophobic drugs in the hydrophobic core, which is conducive to their delivery [135,136]. In addition, micelles have specific targeting effects. Through specific identification and combination with the target group, micelles can target and deliver the drugs to the lesion site, enhance the aggregation of drugs in the lesion site, and reduce the toxicity and side effects on the surrounding normal tissues as much as possible. Moreover, micelles with stimulation response performance can release drugs under certain pH values, temperature, and other stimulation conditions to achieve treatment. Some tumors are in an acidic microenvironment and a low pH value can promote the release of therapeutic drugs at the targeted site. Therefore, the therapeutic effect of micelle-based drug delivery systems can be enhanced [135,136,137]. 

The application of micelle-based drug delivery systems can enhance the efficacy of drugs and reduce their side effects on normal tissues. Ji et al. developed dual-responsive polymeric micelles DOX&ALS@MFM to achieve the synergistic treatment of tumor chemotherapy, PTT, and PDT. The drug-loading micelle system enhanced drug aggregation at the tumor site via folic acid targeting and EPR effect. DOX had a chemical killing effect on tumor cells, while ALS not only converted light energy into heat energy, but also produced singlet oxygen, which killed tumor cells through PTT and PDT. Studies have shown that DOX&ALS@MFM micelles can increase the local temperature at the tumor site and have a good inhibitory effect on tumor growth under NIR light irradiation [138].

To improve drug loading, Huang and his colleagues developed biodegradable polyionic micelles, and then introduced the photothermal material black phosphorus and loaded chemotherapeutic drug Paclitaxel to prepare a therapeutic nanoplatform (PD-M@BP/PTX). The particle size of PD-M@BP/PTX was 124–162 nm, with an encapsulation efficiency of more than 94% and excellent colloidal stability. In vivo, PD-M@BP/PTX not only significantly inhibited the proliferation of tumors, but also reduced the potential damage of chemotherapy drugs to the whole body [139].

## 7. Conclusions and Outlooks

Compared with traditional treatment methods (surgery, chemotherapy, radiotherapy, and combined treatment), PTT based on biological nanomaterials has shown significant advantages in the field of tumor treatment, such as their minimally invasive nature, low toxicity, reduced side effects, and strong tumor cell killing effect. This review focused on the field of OSCC and summarized the latest application progress of biomaterials for photothermal therapy of oral cancer. At present, there are four kinds of biological nanomaterials used for PTT of oral cancer: noble metal nanomaterials, carbon-based nanomaterials, metal compounds, and organic nanomaterials. We summarized the excellent performance and clinical application limitations of each type of materials, hoping to point out the direction for their future improvement.

Although biocompatibility and anti-tumor effects in animal models of PTT were realized, its clinical transformation is an insurmountable challenge. With the development of nanotechnology, novel photothermal nanomaterials emerge endlessly. However, each type of nanomaterials has its limitations in clinical application, especially the long-term safety issue. Noble metal nanomaterials, carbon-based nanomaterials, and metal compounds are used as inorganic nanomaterials. Among various noble metal nanomaterials, gold-based nanomaterials are one of the most explored photothermal nanomaterials for OSCC, while other noble metal nanomaterials are rarely used (such as silver, platinum, and palladium nanomaterials). Gold-based nanomaterials have strong NIR light absorption capacity and high photothermal conversion capacity. However, their production cost is high, and their biocompatibility and degradability are poor. Their potential long-term toxicity and side effects in the human body need to be determined through long-term observation. In addition, carbon-based nanomaterials have good electrochemical properties, strong non-covalent bonding properties, a large surface absorption area, and easy surface modification. However, their photothermal conversion ability and dispersibility in water needs to be improved. Metal compounds have broad application prospects in the field of PTT because of their low cost, good photothermal stability, and low cytotoxicity. Compared with various inorganic nanomaterials, organic nanomaterials are much safer because of their good biocompatibility and good biodegradability. However, sometimes their rapid degradation leads to a very short residence time in the human body, which can result in a poor photothermal treatment effect. Thus, all types of photothermal nanomaterials need in-depth research to obtain more satisfactory biosafety, degradability, and photothermal conversion performance to allow their application in tumor treatment practice. Moreover, the relatively shallow penetration ability of NIR light also limits the clinical transformation of PTT. In recent years, the light in the second near-infrared region (NIR-II, 1000–1700 nm) with deeper penetration depth has been favored by researchers and is expected to provide a solution to this problem [140]. 

Some photothermal agents have a poor aggregation ability in the tumor area, and low photothermal conversion efficiency for different reasons. They can be combined with antibodies such as anti-EGFR antibodies [48] and cancer cell membranes [56]. Some photothermal nanomaterials have high toxicity and a poor therapeutic effect. These inherent defects can be improved, their toxicity reduced, and their photothermal conversion ability improved through physical or chemical modification, and combination with functional groups. Moreover, PTAs can be loaded into a drug delivery system to improve the tumor targeting ability of nanomaterials and the drug concentration at the lesion site, which is more conducive to the elimination of tumors. Lastly, PTT can also be combined with chemotherapy [108], radiotherapy [81], immunotherapy [113], and photodynamic therapy [82] to achieve synergistic therapy. The therapeutic effect of combined therapy on oral cancer is significantly better than that of photothermal therapy alone.

In conclusion, the clinical application of photothermal nanomaterials for OSCC is limited, and further research into their improvements is required. We hope that this summary of the existing photothermal nanomaterials for OSCC and the analysis of their advantages and disadvantages provides ideas and directions for the future research of PTT in OSCC and expands the application space of photothermal nanomaterials.

## Figures and Tables

**Table 1:** A summary of noble metal nanomaterials for PTT in OSCC.

Classification	Photothermal Nanomaterial	Exposure Conditions	Tumor Type	Therapy Model	Ref.
Gold nanorods	UCP@SiO_2_-NR-FA	980 nm	OECM-1 cells	PTT	[54]
	GNRs-siRNA	810 nm	Cal-27 cells	PTT	[39]
	PDDAC-GNRs	780 nm	NT8e cells	PTT	[47]
	EGFR antibody Conjugated gold nanoparticles	514 nm	HSC 313, HOC 3 Clone 8 and HaCaT cells	PTT	[48]
	CO-GNRs	780 nm	Cal-27 cells	PTT	[55]
	GNR@Mem	X-rays, 4 Gy; 980 nm	KB cells	PTT; RT	[56]
	RB-GNRs	PTT: 810 nm; PDT: 532 nm	Cal-27 cells	PTT; PDT	[57]
	NR-HS-Ce6-DOX	665 nm	Cal-27 cells	PTT; PDT; chemotherapy	[58,59]
	DOX-AuNRs@mSiO_2_-HA	808 nm	Cal-27 cells	PTT; chemotherapy	[60]
Gold nanospheres	Apt-HAuNS	-	OSC-19 cells	PTT	[61]
	C225-SPIO@Au NS	808 nm	A431, FaDu, OSC-19 and HN5 cells	PTT	[40]
Others	gold nanoflower		TCA-8113 cells	PTT	[62]
	AuNF@SiO_2_@mSiO_2_	808 nm	Cal-27 cells	PTT	[63]
	^177^Lu-DTPA-pAuNS	793 nm	SAS-3R cells	Brachytherapy; PTT	[41]
	E-GNS	808 nm	KB cells;	PTT	[64]
	tNAs-cisPt	808 nm	SCC-25 cells; UPCI:SCC-154 cells	PTT; chemotherapy	[65]
	Branched gold nanostructures	808 nm	KB cells	PTT	[66]
	E72-Chitosan-Ag_3_AuS_2_	808 nm	Cal-27 cells	PTT	[67]

**Table 2:** A summary of carbon-based nanomaterials and metal compounds for PTT in OSCC.

Classification		Photothermal Nanomaterial	Exposure Conditions	Tumor Type	Therapy Model	Ref.
Carbon-based nanomaterials	Graphene nanomaterials	Fe_3_O_4_@Au/rGO NSs	X-rays, 2 and 4 Gy; 808 nm	KB cells	RT; PTT	[81]
		CPGA	808 nm	SCC7 cells	PTT	[83]
	Carbon nanotubes	ICG-HANP/SWCNT	808 nm	SCC7 cells	PTT; PDT	[82]
	Others	NCOD-HCS	980 nm	FaDu and HaCaT cells	PTT	[84]
Metal compounds	Iron	Fe_3_O_4_@CMCTS	808 nm	KB cells, MCF-7 and S180 cells	PTT	[85]
		MUA-PEG/SiNC/IOC-Au NCPs	NIR light	KB-3-1 and SK-BR-3 cells	PTT; PDT	[86]
	Copper	Cu_2−x_S-RB@DMSN-AE105	laser: 1064 nm; US: 1.0 MHz	OCS-19	PTT; SDT	[87]
		Cu_2−x_S@MnS	808 nm	HeLa cells and HNSCC patient derived xenograft models	PTT; PDT	[88]
		CuO-NPs@L-PLGA/PDA/PEG	808 nm	Cal-33 cells	PTT	[89]
	Molybdenum	MoP_2_ NRs	808 nm	Cal-27, HOK and SCC9 cells	PTT; Chemodynamic therapy	[90]
		Cys-MoO_3−x_ NPs	808 nm	OSCC cells	PTT	[91]

**Table 3:** A summary of organic nanomaterials for PTT in OSCC.

Classification	Photothermal Nanomaterial	Exposure Conditions	Tumor Type	Therapy Model	Ref.
NIR dyes	PEG-PCL-C3-ICG NPs	808 nm	HSC cells	PTT; PDT	[106]
	AuNFs@SiO_2_@mSiO_2_-ICG	-	Cal-27	PTT; PDT	[107]
	DOC-SINPs	808 nm	SCC-15	PTT; chemotherapy	[108]
	CPCI/DOX-NP	808 nm	OSC-3	PTT; chemotherapy	[104]
	SDF-1/ICG/PFH/DOX/PLGA NPs	808 nm	SCC-15	PTT; chemotherapy	[109]
	SDF-a/ICG/PNE/ADRPLGA NPs	-	SCC-15	PTT; chemotherapy	[110]
	HSA-ICG-DDP NPs	808 nm	HSC and CAF cells	PTT; PDT; chemotherapy	[111]
	M@PPI-siRNA	808 nm	SCC-25	PTT; PDT	[112]
	Gel-N-ICG	808 nm	Cal-27 and HIOEC cells	PTT; immunotherapy	[113]
	IR820-loaded gel-MSNs	808 nm	Cal-27	PTT; chemotherapy	[114]
	Porphysomes	808 nm	buccal cancer model	PTT	[115]
Conductive polymers	DOX/Ppy NW	810 nm	KB cells and MCF7 cells	PTT; chemotherapy	[105]
	HATb–PDA–DOX	808 nm	Cal-27, HSC-3 and HGF cells	PTT; chemotherapy	[3]
	HMPBs&DOX@HAMNs	-	OSCC cells	PTT; chemotherapy	[116]

## Data Availability

Not applicable.
